# Calendar

**DOI:** 10.1111/iwj.14023

**Published:** 2022-11-28

**NORTH AMERICA**

**28‐29 January 2023**

***International Conference on Wearable Biomedical Sensors and Devices (ICWBSD)***

**New York, USA**

www.waset.org

**26‐30 April 2023**

***Symposium on Advanced Wound Care (SAWC) Spring 2023***

**National Harbour, MD, USA**

**EUROPE**

**21‐22 January 2023**

***International Conference on Advances in Wound Healing Systems (ICAWHS)***

**Amsterdam, Netherlands**

www.waset.org/conferences-in-january-2023-in-amsterdam/program

**23‐24 January 2023**

***10th International Congress on Infectious Diseases***

**Barcelona, Spain**

www.conferenceseries.com/infectious-diseases-meetings

**2‐3 February 2023**

***Norwegian Wound Care Society***

**Oslo, Norway**

www.nifs-saar.no/oslo-2023

**April 18, 2023**

***WCS Wound Congress 2023***

**Utrecht, The Netherlands**

www.wcs.nl/congres/wcs-wondcongres-2023

**INTERNATIONAL**

**11‐12 January 2023**

***International Conference on Wound Care and Wound Management (ICWCWM)***

**Singapore, Singapore**

https://waset.org

**23‐24 February 2023**

***7^th^ World Congress on Wound Healing and Critical Care***

**Dubai, United Arab Emirates**

www.aast.org

**25‐26 March 2023**

***International Conference on Wound Healing Systems and Technologies (ICWHST*** ***)***

**Tokyo, Japan**

www.waset.org

